# Accuracy of whole-body diffusion-weighted MRI (WB-DWI/MRI) in diagnosis, staging and follow-up of gastric cancer, in comparison to CT: a pilot study

**DOI:** 10.1186/s12880-021-00550-2

**Published:** 2021-02-05

**Authors:** Sofie De Vuysere, Vincent Vandecaveye, Yves De Bruecker, Saskia Carton, Koen Vermeiren, Tim Tollens, Frederik De Keyzer, Raphaëla Carmen Dresen

**Affiliations:** 1 Department of Radiology, University Hospitals Leuven, KU Leuven, Herestraat 49, 3000 Leuven, Belgium; 2 Department of Radiology, Imelda Hospital Bonheiden, Imeldalaan 9, 2820 Bonheiden, Belgium; 3 Department of Gastroenterology, Imelda Hospital Bonheiden, Imeldalaan 9, 2820 Bonheiden, Belgium; 4 Department of Surgery, Imelda Hospital Bonheiden, Imeldalaan 9, 2820 Bonheiden, Belgium

**Keywords:** WB-DWI/MRI, Diffusion-weighted imaging (DWI), Magnetic resonance imaging (MRI), Computed tomography (CT), Stomach neoplasm, Neoplasm metastases, Neoplasm staging, Peritoneal neoplasms

## Abstract

**Background:**

Accurate staging of patients with gastric cancer is necessary for selection of the most appropriate and personalized therapy. Computed tomography (CT) is currently used as primary staging tool, being widely available with a relatively high accuracy for the detection of parenchymal metastases, but with low sensitivity for the detection of peritoneal metastases. Magnetic resonance imaging (MRI) with diffusion-weighted imaging (DWI) has a very high contrast resolution, suggesting a higher diagnostic performance in the detection of small peritoneal lesions. The aim of this study was to retrospectively evaluate the added value of whole-body diffusion-weighted MRI (WB-DWI/MRI) to CT for detection of peritoneal carcinomatosis (PC) and distant metastases in the preoperative staging of gastric cancer.

**Methods:**

This retrospective study included thirty-two patients with a suspicion of gastric cancer/recurrence, who underwent WB-DWI/MRI at 1.5 T, in addition to CT of thorax and abdomen. Images were evaluated by two experienced abdominal radiologists in consensus. Histopathology, laparoscopy and/or 1-year follow-up were used as reference standard.

**Results:**

For overall tumour detection (*n* = *32*), CT sensitivity, specificity, positive predictive value (PPV) and negative predictive value (NPV) was 83.3%, 100%, 100% and 82.4% respectively. For WB-DWI/MRI these values were 100%, 92.9%, 94.7% and 100%, respectively. For staging (*n* = *18*) malignant lymph nodes and metastases, CT had a sensitivity, specificity/PPV/NPV of 50%/100%/100%/71.4%, and 15.4%/100%/100%/31.3% respectively. For WB-DWI/MRI, all values were 100%, for both malignant lymph nodes and metastases. WB-DWI/MRI was significantly better than CT in detecting tumour infiltration of the mesenteric root, serosal involvement of the small bowel and peritoneal metastases for which WB-DWI/MRI was correct in 100% of these cases, CT 0%.

**Conclusions:**

WB-DWI/MRI is highly accurate for diagnosis, staging and follow-up of patients with suspected gastric cancer.

## Background

Gastric cancer remains one of the deadliest neoplasms in the world, with a 5-year survival rate of approximately 25% for all stages [1, 2]. Once spread to the peritoneum, the 5-year survival rate drops to less than 5% [3]. Up to 40% of gastric cancer patients develop some degree of peritoneal metastases during the course of their disease [3]. After gastric cancer resection, haematogenous recurrence is most common (54%), with peritoneal disease being the second most common site of recurrence, accountable for around 43 to 45.9% [4, 5]. Other common sites of recurrence are lymph nodes (12%) and locoregional in 22% [4]. Accurate staging of all tumour locations is essential in these gastric cancer patients to select the best treatment with the highest chance of cure. As these patients have a high risk of peritoneal dissemination, an imaging technique that can detect small volume disease is needed.

According to ESMO Guidelines, outlined by Lerut et al. [6], local staging with gastroscopy and Endoscopic Ultrasound (EU) should be combined with distant staging by a CT scan of the thorax and abdomen to detect metastases elsewhere in the body [6–8]. Sensitivity of CT for the evaluation of lymph node metastases is variable (62.5–91.9%) [9], due to the lack of standard criteria for diagnosing metastatic lymph nodes [10]. Size is the most commonly used criterion to define whether or not a lymph node is metastatic. However, short-axis cut-off levels to define a metastatic lymph node vary between different studies from > 5 to > 10 mm. Furthermore, large lymph nodes may be inflammatory and small lymph nodes may harbour millimetric tumour metastases [11]. The accuracy of CT in detecting metastatic disease to liver and lung, according to Seevaratnam [12] is high (81%). However, a recent review of four studies revealed rather low and variable sensitivities (14.3–59.1%), with high specificities (93.3–99.8%) [13], questioning its standard use in clinical practice. Moreover, CT estimation of peritoneal cancer spread is far from optimal with a sensitivity of only 28.3% (although with a high specificity of 98.9%) [14], due to its limited soft tissue contrast resolution [15, 16]. With its ability to determine the metabolic activity of tissues ^18^F-Fluoro-deoxyglucose positron emission tomography (FDG-PET/CT) is a powerful staging method for oncological patients and is often used in different tumour types with known high FDG avidity, such as lymphoma, melanoma, oesophageal cancer and cervical cancer. However, the variable physiological FDG uptake in the stomach and the low FDG avidity of different gastric cancer types might complicate the evaluation of gastric cancer by PET/CT. Moreover, the value of PET/CT is limited in the assessment of small lymph nodes [17, 18] and especially in the estimation of peritoneal cancer spread, most profoundly in small volume disease [19, 20]. Therefore, both CT and PET/CT are not ideal for accurate staging and treatment planning in gastric cancer [21].

Diagnostic laparoscopy is recommended for all stage IB-III, potentially resectable gastric cancer patients to exclude (PET/)CT-graphically occult metastatic disease [6–8]. A review of 15 studies reported a variable high laparoscopic sensitivity ranging from 64.3 to 94% [22] for the detection of peritoneal metastases, which is higher than in CT. However, laparoscopy remains an invasive surgical procedure and only provides information about structures in the peritoneal cavity. Diffusion-weighted magnetic resonance imaging (DWI/MRI) might solve this challenge. In DWI, contrast is generated between tissues due to differences in water molecule mobility. In highly cellular tissues such as tumours, water diffusion is restricted, resulting in higher signal intensity on DWI with high *b* values (where a high degree of diffusion weighting is applied) and lower apparent diffusion coefficients (ADC), compared to the normal surrounding tissue, where water molecules are less restricted. Due to the high contrast resolution in DWI tumour depiction can significantly be improved, particularly for peritoneal disease [23, 24]. DWI/MRI can be used as a whole body (WB) imaging technique, for evaluation of primary tumour, lymph nodes and metastases in one single, noninvasive examination [25–28]. The aim of this study was to retrospectively evaluate the added value of whole-body diffusion-weighted MRI (WB-DWI/MRI) to CT for detection of peritoneal carcinomatosis (PC) and distant metastases in the preoperative staging of gastric cancer.

## Methods

### Patients

This retrospective study was approved by the institutional review board (Ethical Committee Imelda Hospital Bonheiden). Informed consent was waived. Between November 2015 and April 2019, 32 consecutive patients with suspected primary or recurrent gastric cancer underwent a CT of the thorax and abdomen, for diagnosis and/or assessment of operability. They all underwent an additional WB-DWI/MRI within 20 days of the CT.

### Computed tomography

Patients received oral contrast (30 ml iodinated contrast agent (Telebrix Gastro, Guerbet), 300 mg/ml, in 900 ml water), during 1 h prior to a breath-hold CT scan of the thorax and abdomen (Somatom Force, Siemens Medical Systems, Erlangen, Germany). Images were obtained approximately 70 s following intravenous iodinated contrast injection (80 ml, Xenetix, Guerbet), which is the optimal timing for evaluation of the gastric tumour as well as possible hepatic metastases [10]. Images were acquired in the axial plane, and reconstructed in coronal and sagittal planes (2 mm).

The following parameters were used: pitch 0.6, rotation speed 0.5 s, 1 mm slice thickness, 0 mm slice gap and 0.6 mm collimation. A reference current of 81 mAs (thorax) and 110 mAs (abdomen) was used with automated Care Dose software. CT was acquired with a 512 × 512 matrix and a FOV ranging between 350 and 420 mm depending on patient size, leading to a pixel resolution of 0.68–0.82 × 0.68–0.82 mm. Total examination time was 5 min.

### WB-DWI/MRI

Patient preparation consisted of drinking one litre of pineapple juice during two hours prior to the examination, to minimize the high signal intensities of the bowel content on the diffusion-weighted images. Antispasmodic medication (butylhyoscine, 20 mg IV) was injected at the start of the examination to decrease bowel movement. All WB-DWI/MRI examinations were performed on a 1.5 T scanner (Aera, Siemens Medical Systems, Erlangen, Germany) with multichannel phased-array surface coils and parallel imaging techniques, providing both functional information (offered by DWI) and detailed anatomical information (offered by T2-weighted and contrast enhanced images). Acquisition of the diffusion-weighted images started with a short T1 inversion recovery (STIR) prepulse for background suppression. The images were acquired in the axial plane, free-breathing in four imaging stations (head/neck, thorax, abdomen and pelvis) at *b* = 50 and *b* = 1000 s/mm^2^ (b1000), and reconstructed in the coronal plane from head to femora. Free-breathing coronal single-shot fast spin-echo T2-weighted images were acquired in the four imaging stations. Breath-hold fat-suppressed T1-weighted gradient-echo sequences after the injection of Gadolinium (Dotarem, Guerbet, Roissy, France) were acquired in the axial plane in the four imaging stations and in the coronal plane for the abdomen and pelvis. Mobiview images were automatically reconstructed from the MRI scanner in the axial and coronal plane. Total examination time was 38 min. The detailed sequence parameters of the WB-DWI/MRI can be found in Table 1.

**Table 1:** Detailed sequence parameters of WB-DWI/MRI

	DWI		T2 HASTE	Contrast-enhanced 3D GE	
	Transverse	Coronal	Coronal	Transverse	Coronal
Image stations	4	MPR	3	4	1 (abdomen)
Respiration	Free breathing		Free breathing	3 × 14 s breath hold	12 s
Fat suppression	STIR (IR = 180 ms)		None	Quick fs	Quick fs
*b*-values (s/mm^2^)	50–1000		None	None	None
Parallel imaging factor	2		2	2	2
Repetition time (TR) (ms)	6622		1500	3.65	4.32
Echo time (TE) (ms)	58		87	1.77	1.98
Slice thickness (mm)	5	5	5	3	3
Slice number	43/station		60/station	112/104/88/88	60
Intersection gap (mm)	0		0	0	0
Field of view (FOV) (mm)	430 × 349		400 × 500	400 × 300	450 × 337
Acquired pixel size (mm)	3.36 × 3.36		1.56 × 1.56	1.79 × 1.25	2.01 × 1.41
Reconstructed pixel size (mm)	1.68 × 1.68		1.56 × 1.56	0.63 × 0.63	0.7 × 0.7
Number of signal averages (NSA)	1		1	1	1

### Evaluation of imaging

All 32 patients underwent both CT and WB-DWI/MRI. CT and WB-DWI/MRI-examinations were read by two abdominal radiologists (13 years and 20 years of experience in oncological imaging) in consensus, for identification of the primary tumour, possible lymphadenopathies, peritoneal and/or distant metastases. The images were read in a random order, with a time interval of two months between the interpretation of the CT and the MRI. The readers were blinded to the results of the other imaging technique, patient history, surgical and pathological outcome.

On the CT images, the following criteria were used for metastatic lymph nodes: short axis diameter of > 6 mm, roundness, heterogeneous contrast-enhancement and/or central necrosis. Peritoneal metastases were diagnosed in the presence of nodules on the peritoneal surface, mesentery and/or bowel wall, and/or irregular peritoneal and/or serosal thickening with enhancement. In the presence of ascites, irrespective of nodular peritoneal thickening, a suspicion for peritoneal implants was made. Distant metastases were diagnosed in the presence of malignant lesions in the liver, lungs, bones and/or other organs.

At WB-DWI/MRI, the information of *b*1000 diffusion-weighted images and anatomical sequences were combined. Lymph nodes with markedly higher *b*1000 signal intensity than the surrounding lymph nodes in combination with round and/or irregular shape and heterogeneous signal on T2 and/or post contrast images, were considered to be malignant. For diagnosing peritoneal and distant metastases the same anatomical criteria were used as in CT, in combination with contrast enhancement and/or high signal at b1000 images and a low signal on the ADC map.

For the selection of the operability of the patients, ESMO guidelines were used. Patients were considered operable in the absence of inaccessible lymph nodes, distant mesenteric, distant peritoneal and/or parenchymal metastases.

### Statistical analysis

Sensitivity, specificity, positive predictive value (PPV) and negative predictive value (NPV) of CT and WB-DWI/MRI were calculated for overall tumour detection, staging of malignant lymph nodes and metastases as well as operability assessment. Reference standard for CT and MRI was histopathology, laparoscopy and/or imaging follow-up for at least 1 year. Comparison of CT and MR accuracies was performed using McNemar’s tests, with a *p* value 0.05 indicating a statistically significant difference.

## Results

### Patient and tumour characteristics

The study population consisted of 22 men and 10 women, with an age range of 29–85 years. Eighteen patients were diagnosed with gastric cancer (primary cancers *n* = *15*, recurrences *n* = *3*), all were histopathologically confirmed (adenocarcinoma *n* = *9*, adenocarcinoma with signet ring cell differentiation *n* = *9*). Two patients had gastritis, 12 had a history of gastric cancer with negative follow-up examinations (Table 2).

**Table 2:** Patient and tumour characteristics

N = 32	
Age range (years)	29–85
Gender	
Male	22
Female	10
Gastritis	2
Post gastrectomy	12
Gastric cancer	18
Primary	15
Recurrence	3
Adenocarcinoma	9
Adenocarcinoma with signet ring cell	9
Inoperable	10
Mesenteric root infiltration	6
Serosal small bowel infiltration	5
Peritoneal metastases	6
Inaccessible lymph nodes	3
Brain metastases	1
Bone metastases	2

### Tumour detection

Of the 15 primary cancers and 3 recurrences, WB-DWI/MRI allowed for the detection of tumour in all these 18 patients and CT in 15 patients.

Twelve patients had a history of gastrectomy. CT as well as WB-DWI/MRI showed no disease recurrence. This was confirmed by a negative follow-up for at least 1 year.

Two patients had histopathologically confirmed gastritis. MRI enabled correct diagnosis in 1 patient and a wrong diagnosis in 1 patient (operable gastric cancer). In both patients, no tumour was found on CT.

For tumour detection, WB-DWI/MRI demonstrated a sensitivity of 100%, specificity of 92.9%, PPV of 94.7% and NPV of 100% (18 TP, 13 TN, 1 FP and 0 FN). The numbers for CT were: 83.3%, 100%, 100% and 82.4% (15 TP, 14 TN, 0 FP and 3 FN), respectively. No significant difference in accuracy was found between WB-DWI/MRI and CT (96.9% vs 90.6%, *p* = 0.32).

### Staging of patients with tumour (n = 18)

#### Lymph nodes

Eight patients were diagnosed with adenopathies (locoregional n = 5, retroperitoneal n = 2, paracardial n = 1), 10 without. WB-DWI/MRI enabled detection of the adenopathies in all patients, on CT lymphadenopathies were missed in 4 patients (locoregional n = 3 and paracardial n = 1), but were correctly interpreted in the other patients (sensitivity 50%, specificity 100%, PPV 100%, NPV 71.4%). WB-DWI/MRI demonstrated a significantly higher accuracy than CT (100% vs 77.8%, *p* = 0.046).

#### Distant metastases

**Fig. 1:**
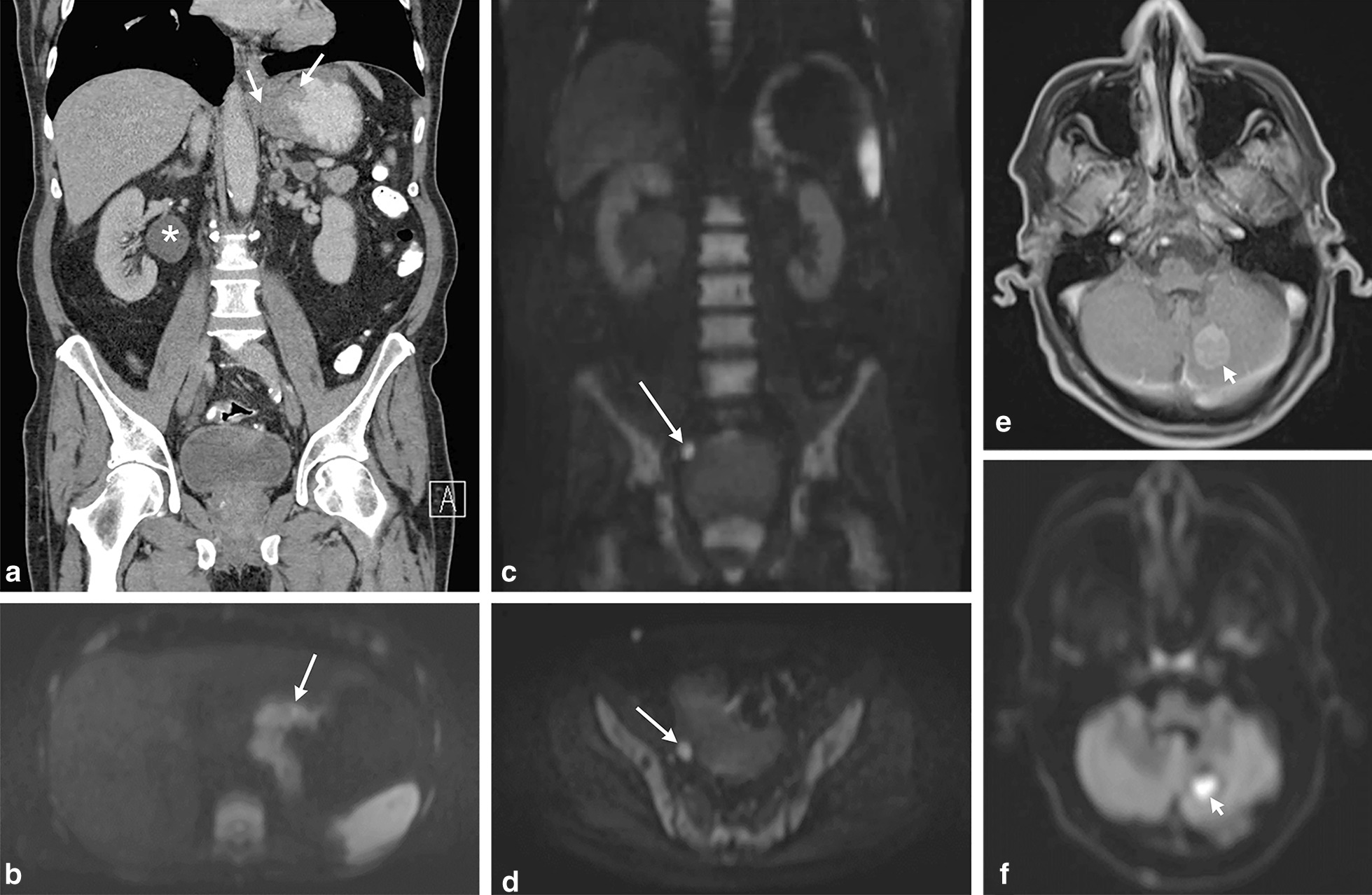
A patient with an age-range between 60 and 70 years old was diagnosed with a primary gastric adenocarcinoma with signet ring cell differentiation, localized at the cardia. CT scan (**a**) showed the primary tumor (arrows) as well as hydronephrosis of the right kidney (*), without a demonstrable cause. He had a WB-DWI/MRI for operability assessment. The primary gastric cancer is well visualized on the axial b1000 DWI (**b**, arrow). Apart from a peritoneal tumor implant on the right distal ureter (arrows) as the cause for the hydronephrosis on the coronal (c) and axial b1000 DWI (**d**), a brain metastasis (arrowheads) was found in the left cerebellum on the axial post-contrast T1 image (**e**) as well as the b1000 DWI (f), since the brain is included in the WB-DWI/MRI

Thirteen patients were diagnosed with (a combination of different) distant metastases (peritoneal n = 13, bone n = 2, brain n = 1), 5 without. Figure 1 is an example of a patient with both peritoneal and brain metastases. The presence or absence of metastases was correctly identified on WB-DWI/MRI in all patients (sensitivity, specificity, PPV and NPV 100%). CT allowed for the detection of peritoneal metastases in only 2 of the 13 patients, the bone and brain metastases were not identified on CT (on a per-patient basis: sensitivity 15.4%, specificity 100%, PPV 100%, NPV 31.3% (2 TP, 5 TN, 0 FP, 11 FN). Accuracy on WB-DWI was significantly higher than on CT (100% vs 38.9%, *p* < 0.001).

### Operability assessment

**Fig. 2:**
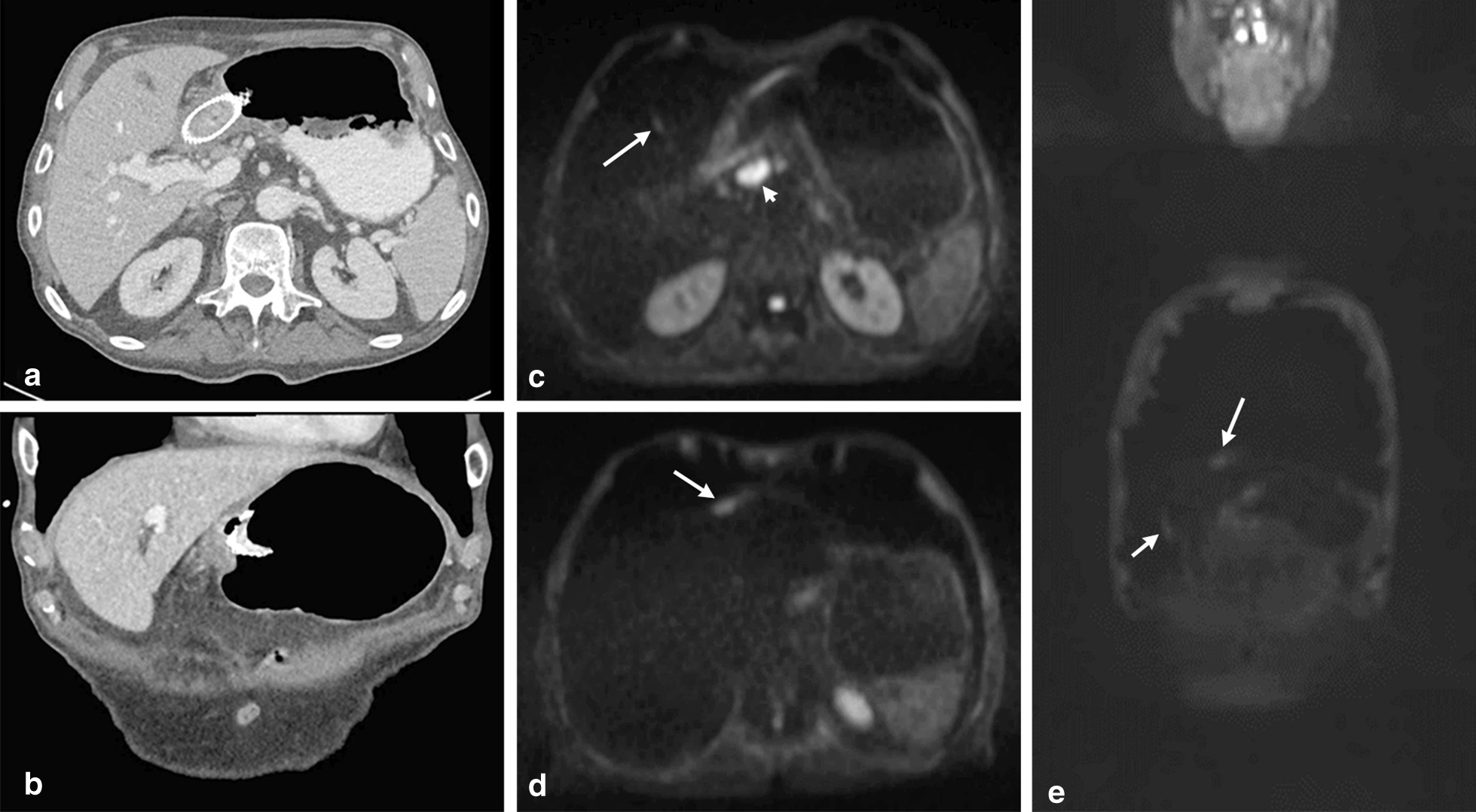
A patient with an age-range between 70 and 80 years old was diagnosed with primary gastric cancer, for which an endoluminal stent was placed. CT scan in the axial plane (**a**) with coronal reconstruction (**b**) did not show any metastases. WB-DWI/MRI was performed for operability assessment, revealing an adenopathy in the gastro-hepatic fat (**c**, arrowhead), as well as peritoneal metastasis in the falciform ligament and on the surface of the left liver lobe (**c**–**e**, arrows)

**Fig. 3:**
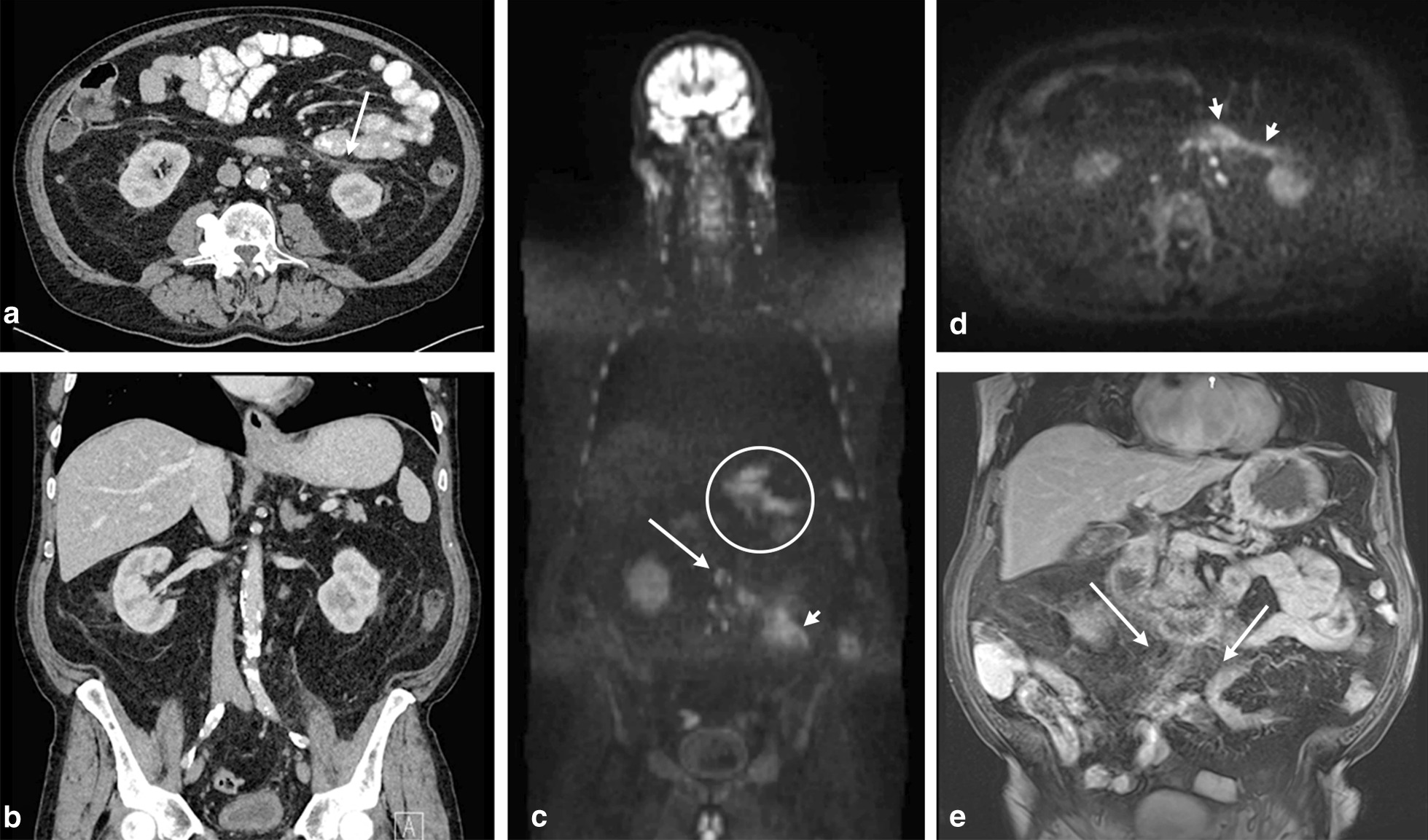
A patient with an age-range between 70 and 80 years old was diagnosed with primary gastric cancer. CT scan in the axial plane (**a**) with coronal reconstruction (**b**) did not show distant metastases. However, a slightly thickened anterior pararenal fascia on the left side (**a**, arrow) was noticed. WB-DWI/MRI was performed for further investigation with coronal (**c**) and axial b1000 (**d**) as well as contrast-enhanced T1-weighted images (**e**), and revealed –apart from the primary tumor (circled)– mesenteric tumor spread along the mesenteric root (**c**–**e**, arrows), as well as peritoneal tumor spread on the left anterior pararenal fascia (**c**, **e**, arrowheads)

**Fig. 4:**
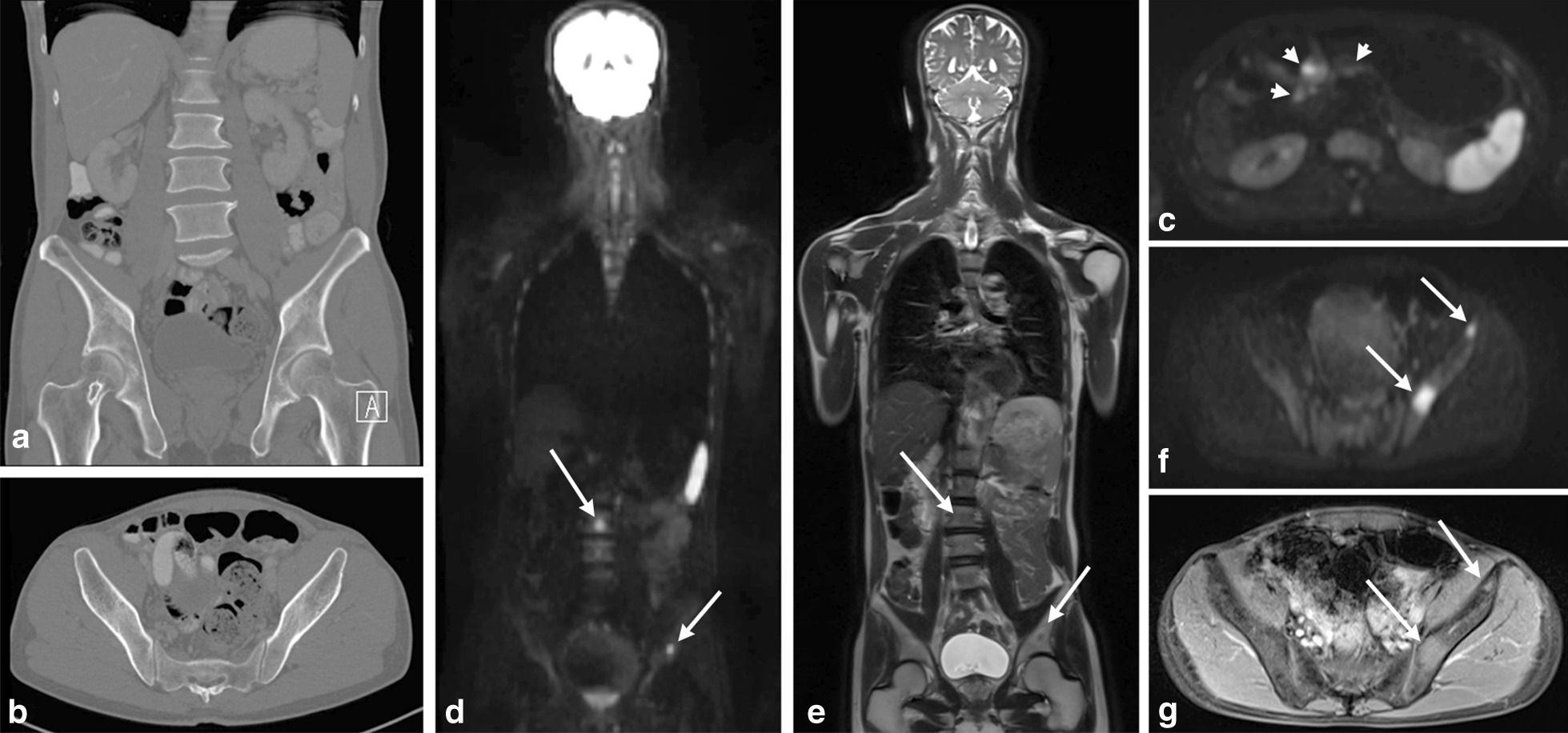
A patient with an age-range between 60 and 70 years old with a primary gastric cancer, where CT did not show any distant metastases (**a**, **b**), underwent a WB-DWI/MRI for operability assessment. Axial b1000 DWI images revealed millimetric tumor implants on the pancreatic surface (**c**, arrowheads). At the same time, multiple bone metastases (arrows) could be seen on the coronal b1000 DWI (**d**), coronal T2-weighted images (**e**), axial b1000 DWI (**f**) and post-contrast T1-weighted images (**g**)

Of the 18 patients with tumour, 10 patients turned out to be inoperable and 8 patients were operable. Figures 2, 3 and 4 show several examples of patients with metastatic disease spread, either still operable (Fig. 2) or inoperable (Figs. 3 and 4), depending on metastatic location and extent. All three recurrences were inoperable. Two of them had peritoneal (serosal) metastases, not visible on CT, with secondary small bowel obstruction. The third patient had inaccessible retroperitoneal lymphadenopathies diagnosed on CT, but also peritoneal implants not identified on CT.

Of the 15 primary cancers, 7 were inoperable. In 2 patients, CT enabled correct suggestion of inoperability due to distant lymphadenopathies (n = 2), and hydronephrosis due to possible tumoural obstruction of the distal ureter (n = 1). The other 5 patients seemed operable on CT. However, they were all identified as clearly inoperable on the WB-DWI/MRI examination due to peritoneal metastases (n = 7), brain metastases (n = 1) and bone metastases (n = 2). In 6 patients metastases were confirmed during follow-up imaging; in 1 patient peritoneal metastases were confirmed by histopathology during laparoscopy.

The other 8 patients were able to undergo curative surgery. These patients all seemed primarily operable on CT and WB-DWI/MRI. The added value of WB-DWI/MRI was in the detection of the primary tumour (n = 3) and better delineation of the extent of the tumour spread (n = 3). In three patients (3/8; 37.5%), small peritoneal tumour implants on the surface of the pancreas were found on WB-DWI/MRI, which were not detected by laparoscopy, though histopathologically confirmed after surgery.

Overall, the numbers for prediction of inoperability for CT were: sensitivity of 30%, specificity 100%, PPV 100% and NPV 53.3%. For WB-DWI/MRI all these values were 100%, leading to an accuracy that was significantly higher than CT (100% vs 61.1%, *p* = 0.008).

## Discussion

Patients with gastric cancer/recurrence benefit from accurate diagnosis, staging and follow-up, maximizing the chance of complete resection and improved survival. The present study showed a high accuracy of WB-DWI/MRI of 96.9% for tumour detection, with only one false positive result, where a patient with gastritis was falsely interpreted as a small operable gastric cancer. Furthermore, WB-DWI/MRI was highly accurate for the prediction of inoperability (PPV 100%, NPV 100%), compared to CT (PPV 100%, NPV 53.3%). In 3 patients, WB-DWI/MRI revealed small peritoneal implants on the surface of the pancreas, not detectable with laparoscopy, suggesting higher sensitivity of WB-DWI/MRI over laparoscopy.

According to recent literature [29–32], the role of MRI in gastric cancer imaging has become more important with DWI and the calculation of ADC as a possible biomarker in diagnosis, T-staging and treatment response assessment [29]. Little is written about the role of MRI in determining metastatic gastric cancer [32]. Some studies suggest that the diagnostic performance of (DWI-)/MRI [33, 34] does not significantly differ from ^18^F-FDG PET/CT or CT. However, these studies contained few patients with peritoneal disease (3/49 gastric cancers) [33] and only one gastric adenocarcinoma (out of 30 different gastrointestinal malignancies) [34]. Moreover, both studies used different bowel preparation as well as different *b* values [33, 34].

Other studies involving tumours with different histopathology (mainly ovarian and colorectal) [15, 23, 28] showed that CT had an insufficient performance to detect PC with accuracies around 51–88%, being even higher than in our study with an accuracy of CT of 38.9%. CT especially fails in detecting tumour infiltration of the mesenteric root and serosal involvement of the small bowel with an accuracy in our study of 0%, compared to other studies where accuracies ranged from 21 to 48% [15, 23, 28, 35, 36]. DWI/MRI is known to be very good at detecting peritoneal tumour implants and implants at the small bowel wall with accuracies of 92–95% [23, 28], which is completely in line with our study (100%). Although the diffusion-weighted images are scanned with a 5 mm slice thickness, metastatic deposits of 2–4 mm can still be detected with a good accuracy, because these deposits contain a lot of remaining signal on high b-value images, due to high cellularity and restriction to water proton movement, whereas the signal of nearly all other tissues is strongly suppressed. In a meta-analysis of 67 articles consisting of 145 studies MRI demonstrated greater accuracy than CT or bone scan and comparable accuracy to PET/CT in diagnosis of skeletal metastases. On a per-lesion basis, the sensitivity and specificity of MRI, FDG-PET/CT, CT, and bone scan were 91 and 96%, 94 and 97%, 75 and 94%, and 77 and 83%, respectively [37, 38]. Also in our study, bone metastases were identified by MRI in two patients, not detected by CT. Tunaria N. and colleagues recently reviewed the expanding role of WB-DWI/MRI in oncological practice [39].

A potential limitation of this study is the small study population, presenting both primary and recurrent tumours. However, to our knowledge this is the first study to describe the role of WB-DWI/MRI in gastric cancer, with the confirmation that it also works on 1.5 T and not only 3 T.

## Conclusion

With the results of this pilot study we can conclude that WB-DWI/MRI is a powerful one-stop imaging method in staging gastric cancer, providing all needed information about disease extent and disease location, inside and outside the abdominal cavity, to successfully determine operability. We believe that MRI can thus gradually replace CT for staging gastric cancer with increasing availability of MRI-systems, ongoing technical optimizations that further decrease imaging time and increasing radiologist training and more generalized expertise. The results of this pilot study serve as a baseline for future larger—and preferentially—multicenter trials aimed at further validation of reproducibility and added clinical value with respect to the separate histological tumor types and initial locoregional tumour stages.

## Data Availability

The datasets used and/or analysed during the current study are available from the corresponding author on reasonable request.

## Abbreviations

CT: Computed tomography
MRI: Magnetic resonance imaging
DWI: Diffusion-weighted Imaging
WB-DWI/MRI: Whole-body diffusion-weighted MRI
PC: Peritoneal carcinomatosis
FDG-PET/CT: ^18^F-Fluoro-deoxyglucose positron emission tomography
ADC: Apparent diffusion coefficient

## References

[1] Bray F, Ferlay J, Soerjomataram I (2018). Global cancer statistics 2018: GLOBOCAN estimates of incidence of mortality worldwide for 36 cancers in 185 countries. CA Cancer J Clin.

[2] Jemal A, Bray F, Center MM, Ferlay J, Ward E, Forman D (2011). Global cancer statistics. CA Cancer J Clin.

[3] Sarela AI, Miner TJ, Karpeh MS, Coit DG, Jaques DP, Brennan MF (2006). Clinical outcomes with laparoscopic stage M1, unresected gastric adenocarcinoma. Ann Surg.

[4] Maehara Y, Hasuda S, Koga T, Tokunaga E, Kakeji Y, Sugimachi K (2000). Postoperative outcome and sites of recurrence in patients following curative resection of gastric cancer. Br J Surg.

[5] Yoo CH, Noh SH, Shin DW (2000). Recurrence following curative resection for gastric carcinoma. Br J Surg.

[6] Lerut T, Stordeur S, Verleye L et al. Clinical practice guidelines: upper gastrointestinal cancer—update. Report 179A. Brussels, Belgium: Belgian Health Care Knowledge Centre; 2012.

[7] Smyth EC, Verheij M, Allum W, Cunningham D, Cervantes A, Arnold D, on behalf of the ESMO Guidelines Committee (2016). Gastric cancer: ESMO Clinical Practice Guidelines for diagnosis, treatment and follow-up. Ann Oncol.

[8] Coburn N, Cosby R, Klein L, Knight G, Malthaner R, Mamazza J, Mercer CD, Ringash J (2017). Staging and surgical approaches in gastric cancer: a clinical practice guideline. Curr Oncol.

[9] Kwee RM, Kwee TC (2009). Imaging in assessing lymph node status in gastric cancer. Gastric Cancer.

[10] Choi JI, Joo I, Lee JM (2014). State of the art preoperative staging of gastric cancer by MDCT and magnetic resonance imaging. World J Gastroenterol.

[11] Borggreve AS, Goense L, Brenkman H, Mook S, Meijer G, Wessels F, Verheij M, Jansen E, Van Hillegersberg R, Van Rossum P, Ruurda J (2019). Imaging strategies in the management of gastric cancer: current role and future potential of MRI. Br J Radiol.

[12] Seevaratnam R, Cardoso R, McGregor C (2012). How useful is preoperative imaging for tumor, node, metastasis (TNM) staging of gastric cancer? A meta-analysis. Gastric Cancer.

[13] Kwee RM, Kwee TC (2015). Modern imaging techniques for preoperative detection of distant metastases in gastric cancer. World J Gastroenterol.

[14] Kim SJ, Kim H-H, Kim YH, Hwang SH, Lee HS, Park DJ (2009). Peritoneal metastasis: detection with 16- or 64-detector row CT in patients undergoing surgery for gastric cancer. Radiology.

[15] Koh JL, Yan TD, Glenn D, Morris DL (2009). Evaluation of preoperative computed tomography in estimating peritoneal cancer index in colorectal peritoneal carcinomatosis. Ann Surg Oncol.

[16] Rivard JD, Temple WJ, McConnell YJ, Sultan H, Mack LA (2014). Preoperative computed tomography does not predict resectability in peritoneal carcinomatosis. AM J Surg.

[17] Kim EY, Lee WJ, Choi D, Lee SJ, Choi JY, Kim BT, Kim HS (2011). The value of PET/CT for preoperative staging of advanced gastric cancer: comparison with contrast-enhanced CT. Eur J Radiol.

[18] Park K, Jang G, Baek S, Song H (2014). Usefulness of combined PET/CT to assess regional lymph node involvement in gastric cancer. Tumori.

[19] Lopez-Lopez V, Cascales-Campos PA, Gil J, Frutos L, Andrade RJ, Fuster-Quinonero M (2016). Use of 18F-FDG PET/CT in the preoperative evaluation of patients diagnosed with peritoneal carcinomatosis of ovarian origin, candidatest o cytoreduction and hipec. A pending issue. Eur J Radiol.

[20] Dromain C, Leboulleux S, Auperin A, Goere D, Malka D, Lumbroso J (2008). Staging of peritoneal carcinomatos: enhanced CT vs PET/CT. Abdom Imaging.

[21] Pasqual EM, Bacchetti S, Bertozzi S (2013). Diagnostic accuracy of preoperative CT scan and 18F-FDG PET/CT in patients with peritoneal carcinomatosis undergoing hyperthermic intraperitoneal chemotherapy (HIPEC) following cytoreductive surgery. Eur J Cancer.

[22] Leake P-A, Cardoso R, Seevaratnam R, Lourenco L, Helyer I, Mahar A (2012). A systematic review of the accuracy and indications for diagnostic laparoscopy prior to curative-intent resection of gastric cancer. Gastric Cancer.

[23] Low RN, Barone R, Lucero J (2015). Comparison of MRI and CT for predicting the peritoneal cancer index (PCI) preoperatively in patients being considered for cytoreductive surgical procedures. ANN Surg Oncol.

[24] Low RN (2016). Preoperative and surveillance MR imaging of patients undergoing cytoreductive surgery and heated intraperitoneal chemotherapy. J Gastrointest Oncol.

[25] Michielsen K, Vergote I, Op De Beeck K, Amant F, Leunen K, Moerman P (2014). Whole-body MRI with diffusion-weighted sequence for staging of patients with suspected ovarian cancer: a clinical feasibility study in comparison to CT and FDG-PET/CT. Eur Radiol.

[26] Michielsen K, Dresen R, Vanslembrouck R, De Kezer F, Amant F, Mussen E (2017). Diagnostic value of whole body diffusion-weighted MRI compared to computed tomography for pre-operative assessment of patients suspected for ovarian cancer. Eur J Cancer.

[27] Koh D-M, Takahara T, Imai Y, Collins DJ (2007). Practical aspects of assessing tumors using clinical diffusion-weighted imaging in the body. Magn Reson Med Sci.

[28] Dresen RC, De Vuysere S, De Keyzer F, Van Cutsem E, Prenen H, Vanslembrouck R, De Hertogh G, Wolthuis A, D’Hoore A, Vandecaveye V. Whole-body diffusion-weighted MRI for operability assessment in patients with colorectal cancer and peritoneal metastases. Cancer Imaging (2019); 19(1):1 10.1186/s40644-018-0187-z PMC6322317 30616608

[29] Giganti F, Tang L, Baba H (2019). Gastric cancer and imaging biomarkers: part 1—a critical review of DW-MR and CE-MDCT findings. Eur Radiol.

[30] Tang L, Wang XJ, Baba H, Giganti F (2019). Gastric cancer and image-derived quantitative parameters: part 2—a critical review of DCE-MRI and ^18^F-FDG PET/CT findings. Eur Radiol.

[31] De Cobelli F, Palumbo D, Albarello L, Rosati R, Giganti F (2020). Esophagus and stomach: is there a role for MR imaging?. Magn Reson Imaging Clin N Am.

[32] Borggreve AS, Goense L, Brenkman H, Mook S, Meijer G, Wessels F, Verhey M, Jansen E, Van Hillegersberg R, Van Rossum P, Ruurda J (2019). Review Article: Imaging strategies in the management of gastric cancer: current role and future potential of MRI. Br J Radiol.

[33] Joo I, Lee JM, Kim JH, Shin D-I, Han JK, Choi BI (2015). Prospective comparison of 3T MRI with diffusion-weighted imaging and MDCT for the preoperative TNM staging of gastric cancer. J Magn Reson Imaging.

[34] Soussan M, Des Guetz G, Barrau V, Aflalo-Hazan V, Pop G, Mehanna Z (2012). Comparison of FDG-PET/CT and MR with diffusion-weighted imaging for assessing peritoneal carcinomatosis from gastrointestinal malignancy. Eur Radiol.

[35] Chua T, Al-Zahrini A, Saxena A, Glenn D, Liauw W, Zhao J (2011). Determining the association between preoperative computed tomografphy findings and postoperative outcomes after cytoreductive surgery and perioperative intraperitoneal chemotherapy for pseudomyxoma peritonei. Ann Surg Oncol.

[36] Mazzei M, Khader L, Cirigliano A, Cioffi Squitieri N, Guerrini S, Forzoni B (2013). Accuracy of MDCT in the preoperative definition of peritoneal cancer index (PCI) in patients with advanced ovarian cancer who underwent peritonectomy and hyperthermic intraperitoneal chemotherapy (HIPEC). Abdom Imaging.

[37] Yang HL, Liu T, Wang XM, Xu Y, Deng SM (2011). Diagnosis of bone metastases: a meta-analysis. Eur Radiol.

[38] Liu T, Wang S, Liu H, Meng B, Zhou F, He F, Shi X, Yang H (2017). Detection of vertebral metastases: a meta-analysis comparing MRI, CT, PET, BS and BS with SPECT. J Cancer Res Clin Oncol.

[39] Tunariu N, Blackledge M, Messiou C, Petralia G, Padhani A, Curcean S (2020). What’s new for clinical whole-body MRI (WB-MRI) in the 21st century. Br J Radiol.

